# Genomic communication via circulating extracellular vesicles and long-term health consequences of COVID-19

**DOI:** 10.1186/s12967-023-04552-2

**Published:** 2023-10-10

**Authors:** Soumyalekshmi Nair, Estefania Nova-Lamperti, Gonzalo Labarca, Arutha Kulasinghe, Kirsty R. Short, Flavio Carrión, Carlos Salomon

**Affiliations:** 1 Translational Extracellular Vesicles in Obstetrics and Gynae-Oncology Group, UQ Centre for Clinical Research, Royal Brisbane and Women’s Hospital, Faculty of Medicine, The University of Queensland, Brisbane, Qld 4072 Australia; 2 Molecular and Translational Immunology Laboratory, Clinical Biochemistry and Immunology Department, Pharmacy Faculty, Universidad de Concepción, Concepción, Chile; 3 Frazer Institute, Faculty of Medicine, The University of Queensland, Brisbane, Qld 4102 Australia; 4 School of Chemistry and Molecular Biosciences, The University of Queensland, Brisbane, Australia; 5 Departamento de Investigación, Postgrado y Educación Continua (DIPEC), Facultad de Ciencias de la Salud, Universidad del Alba, Santiago, Chile

**Keywords:** COVID-19, SARS-CoV-2, Metabolism, Long-term consequences

## Abstract

COVID-19 continues to affect an unprecedented number of people with the emergence of new variants posing a serious challenge to global health. There is an expansion of knowledge in understanding the pathogenesis of Coronavirus disease 2019 (COVID-19), caused by severe acute respiratory syndrome coronavirus 2 (SARS-CoV-2), and the impact of the acute disease on multiple organs. In addition, growing evidence reports that the impact of COVID-19 on different organs persists long after the recovery phase of the disease, leading to long-term consequences of COVID-19. These long-term consequences involve pulmonary as well as extra-pulmonary sequelae of the disease. Noteably, recent research has shown a potential association between COVID-19 and change in the molecular cargo of extracellular vesicles (EVs). EVs are vesicles released by cells and play an important role in cell communication by transfer of bioactive molecules between cells. Emerging evidence shows a strong link between EVs and their molecular cargo, and regulation of metabolism in health and disease. This review focuses on current knowledge about EVs and their potential role in COVID-19 pathogenesis, their current and future implications as tools for biomarker and therapeutic development and their possible effects on long-term impact of COVID-19.

## Introduction

Extracellular vesicles (EVs) are stable lipid bilayer nanovesicles released by cells and encapsulate cell specific signalling molecules, which once delivered at proximal or distal target cells, are capable of regulating their function [1]. EVs are attractive liquid biopsy tool as they are packaged with tissue specific signalling molecules including protein, lipids and nucleic acids derived from their parental cell [2]. EVs are a unique source of biomarkers, and potential therapeutics, as their content is stabilised and protected against enzymatic degradation [3, 4]. Since EVs are key regulators of physiological processes in health and disease [5, 6] the role of EVs in regulating the common physiological processes that are dysregulated in acute and long COVID and its utility as diagnostic and therapeutic tools in COVID-19 is an attractive area of discussion.

EVs is an umbrella term and covers a heterogenous population of vesicles of different sizes from around 30 nm to 10 uM, originating from discrete biological pathways [7]. Among these, exosomes (around 30–150 nm) are EVs, which originate from the endosomal pathway through invagination of the plasma membrane to form endosomes. Maturation of late endosomes results in multi-vesicular bodies (MVBs) containing intraluminal vesicles (ILVs), and release of ILVs into the extracellular space results in exosomes [2]. Microvesicles/ectosomes (around 50 nm–1 µm) are formed from external budding of the plasma membrane. Apoptotic bodies (around 50 nm–5 µm) are released by activation of apoptotic signalling [2]. Other types of vesicles include migrasomes (around 500 nm–3 µm), released by migratory cells and large oncosomes (around 1–10 µm), secreted by some cancer cells [7]. With regard to COVID-19, growing evidence suggests the key roles of EVs, including circulating levels of EVs and its cargo in disease pathogenesis [8–12], potential utility for the development of biomarkers [13, 14], treatment options [15–21], and in vaccination and immunity [22–26].

Coronavirus disease 2019 (COVID-19) caused by the severe acute respiratory syndrome coronavirus 2 (SARS-CoV-2), was first reported in Wuhan, China, in December 2019. Due to the rapid spread, COVID-19 was declared a pandemic by the World Health Organization (WHO) in March 2020, as it posed an unprecedented challenge to public health, and the global economy [27]. The clinical characteristics of COVID-19 can range from asymptomatic infection to fatal or life-threatening conditions. Available epidemiological data has shown that the features of the acute disease is a strong predictor of long term effects of COVID [28, 29]. There is a critical need to develop a comprehensive understanding of the long-term impact of SARS-CoV-2 infection on multiple organs in recovered patients, and identify the features of acute disease which can predict long-term consequences. This will provide opportunities to identify people who may develop long-term sequelae, including the type of sequelae, and allow introduction of therapeutic or management strategies in the acute disease which can prevent the long-term consequences of COVID-19.

EVs have shown relevance in COVID-19 pathogenesis, biomarker development, treatment strategies, and immune responses. In the context of COVID-19, understanding the acute disease's impact on long-term consequences is vital for identifying individuals at risk and implementing preventive measures. In this review, we will provide an overview of the patophisiology of SARS-CoV-2 infection, the development of COVID-19 and the potential roles of EVs in this phenomenon.

## Pathophysiology of SARS-COV-2 infection

SARS-CoV-2 is an enveloped, positive-sense, single-stranded RNA virus belonging to the *Coronaviridae* family and with sequence similarity to the SARS-CoV-1 and MERS-CoV (Middle East respiratory syndrome) virus [27]. The structural spike (S) protein in the SARS-CoV-2 viral capsid is responsible for virus-receptor binding [30]. The SARS-CoV-2 S protein binds to the human angiotensin-converting enzyme 2 (ACE2) receptor in cells and the cellular serine protease TMPRSS2 primes the S protein for this interaction. This event triggers a cascade of reactions which facilitates the entry of the virus into the cells, followed by viral transcription and replication [31].

After entry into the cells virus undergoes active replication and release leading to pyroptosis of the cell and release of damage-associated molecular patterns (DAMPs). DAMPs are recognized by neighbouring cells including immune cells such as macrophages triggering pro-inflammatory response. Monocytes, macrophages and T cells are recruited to the site of inflammation promoting further inflammation [32]. In the lungs, accelerating viral infection damages epithelial-endothelial barrier integrity, triggering the influx of inflammatory cells and an acute inflammatory response. This leads to the development of pulmonary oedema and impaired alveolar-capillary oxygen transmission and diffusion capacity, which can often become fatal, causing acute respiratory distress syndrome (ARDS), and multiple organ failure. Most importantly, SARS-CoV-2 infection can cause overactivation of innate immunity and increased secretion of cytokines. This hyperactive immune response characterized by the release of interferons, interleukins, tumour-necrosis factors, chemokines, and several other inflammatory mediators is termed a “cytokine storm”, which is a major complication of COVID-19 [33]. Studies show that rapid clinical deterioration and mortality in COVID-19 patients could be linked to the high blood concentrations of inflammatory biomarkers of the cytokine storm [34–36].

SARS-CoV-2 infection reprograms the proteome and transcriptome in host cells to promote viral life cycle and sustenance in host [37, 38]. Mitochondrial proteins are primary targets for SARS-CoV-2 virus. Mitochondrial apoptosis proteins are upregulated by SARS-CoV-2 in infected cells leading to cell death and organ damage [39, 40]. The viral life cycle in the host cell is intimately associated with the lipid and glucose metabolic pathways [41]. SARS-CoV-2 utilizes the cellular lipid and glucose metabolic pathways for viral entry, replication and egress leading to altered serum lipid and aminoacid profile with abnormal glucose homeostasis [42]. In this way, SARS-CoV-2 reprograms the host system by altering the protemic, transcriptomic, lipidomic and metabolic landscape favouring rapid spread and perpetuation of virus. In most individuals, the host immune response counteracts the SARS-CoV-2 infection and the disease manifests as a mild infection confined to the upper respiratory tract, but in some individuals various immune-pathological factors lead to severe disease characterized by multi-organ damage [43].

## Extracellular vesicles and COVID-19

### Extracellular vesicles and SARS-CoV-2 infection

EVs are released from all cell types including SARS-CoV-2 infected cells, and both EVs and the virus share similarities in terms of size, cellular pathways of biogenesis/infection, cellular release, and uptake by target cells [44, 45]. Many viruses hijack the exosome biogenesis pathway for viral assembly, maturation, trafficking and release, and EVs released from virus infected cells are enriched in viral proteins and nucleic acids [46–49]. During viral replication in host cell, the viral proteins and nucleic acids can be incorporated into the ILVs in MVBs of endosomal compartments and consequently delivered via exosomes [50–52]. In the context of SARS-CoV-2, direct evidence showing the crosstalk between EV biogenesis and viral infectious cycle is not available. However, EVs carrying viral S protein were isolated from blood samples of SARS-CoV-2 infected patients [53]. Cells expressing SARS-CoV-2 S protein releases EVs with surface expression of full-length S protein indicating that S protein can be incorporated in EVs [54]. Interestingly, this study showed that viral S protein in EVs is functional and can act as targets for neutralizing antibodies from commercial and convalescent patient sera in vitro. This suggests that presence of EVs carrying S protein could act as decoys of viral neutralizing antibodies which in turn promote viral entry in cells [54]. However, comparative proteomic characterization of EVs isolated from plasma of patients with mild and severe disease reported higher expression of viral S protein in EVs from mild patients than severe patients [53]. In addition, mild COVID-19 EVs activated CD4+ T helper cells and enhanced immune response against SARS-CoV-2 antigen compared to EVs from severe patients in vitro [53]. Another study identified that S protein carrying EVs were induced following mRNA-based vaccine administration [55]. Furthermore, EVs containing viral S protein elicited S protein specific antibody response following vaccination [55]. This reinforces the ability of EVs to present the viral S protein to antigen processing cells and activate immune response. Hence, the presence of viral S protein in EVs might be a mediator of immune activation or antibody inactivation depending on the complex interplay between EVs, SARS-CoV-2 and the immune system.

Whilst studies have reported the presence of S protein in EVs, it is unclear whether EVs acquire the viral S protein from cells during active viral replication. In addition, EVs in patient plasma might acquire the viral components in extracellular environment or the viral particle contaminants might be copurified with EV isolations. Interestingly, whole SARS-CoV-2 can be packaged into EVs released from cells undergoing virus induced apoptosis [56]. Electron microscopy analysis revealed SARS-CoV-2 induce apoptotic changes in infected cells releasing larger EVs approximately 1–10 µm in diameter containing large numbers of live viral particles [56]. The EVs carrying viral particles protect the virus from neutralizing antibodies and mediate viral entry in target cells by receptor-independent uptake mechanism [56]. Hence EVs can act as vehicles for SARS-CoV-2 to circulate in the system evading immune response and promotes a universal delivery of viruses bypassing specific receptor mediated viral entry. On the other hand, ACE-2 postive EVs were reported to be released by cell and identified to act as decoys for removal of SARS-CoV-2 by binding to the S protein [18, 54, 57]. In this way, heterogenity in the molecular composition of EVs might impart contradictory effects on viral propagation and disease progression.

### Extracellular vesicles as mediators of acute inflammation in COVID-19

Inflammation is a key feature of COVID-19 and severe disease is associated with sudden release of pro-inflammatory cytokines such as IL-1, IL-6, TNF-α and interferon, called ‘cytokine storm’, leading to the migration of immune cells into the infection site, capillary damage, and multi-organ failure [58]. Differential expression of inflammatory and cardiovascular disease related proteins in circulating EVs in COVID-19 has been reported and expression levels of pro-inflammatory proteins, EN-RAGE (extracellular newly identified receptor for advanced glycation and end products binding protein), TF (tissue factor), and IL-18R1 in EVs were correlated to disease severity [59]. The EVs carrying differential expression of inflammatory proteins induced apoptosis in pulmonary endothelial cells in the order of the disease severity of patients [59]. Another study showed an increase in anti-inflammatory metabolites such as LysoPS, 7-α,25-Dihydroxycholesterol, and 15-d-PGJ2 and decrease in coagulation related metabolites such as thromboxane and elaidic acid in EVs from COVID-19 patients [60]. The altered profile of inflammatory proteins in EVs could be an effect of the inflammatory pathology in the cells reflected in the EVs. On the other hand, EVs can play a role in mediating the immune-pathological effects of SARS-CoV-2 in distant cells. This has been shown in vitro by exposing target cells to EVs derived from patients from different severity groups versus healthy controls and identifying an altered inflammatory response in target cells [59, 61]. It was reported that tenascin-C and fibrinogen-β are highly abundant in circulating small EVs from COVID-19 patients in comparison to healthy controls, and that exposure of human hepatocytes to these EVs triggers proinflammatory cytokines, evidenced by an increased expression of TNF-α, IL-6, and CCL5 [61]. Further EVs from severe patients induced NLRP3, IL-1β, and caspase-1, in microvascular and liver endothelial cells [62], and triggered apoptosis and cell death in pulmonary microvascular endothelial cells [59] compared to EVs from patients with mild disease and healthy controls. Also, it has been described that SARS-CoV-2 S protein transfected HEK-293 T cells release EVs with miR-148a and miR-590, which when internalised by human microglial cells, targets the USP33-IRF9 pathway, suggesting an important role as effectors in neuroinflammatory damage [12]. Hence EVs could be mediators of inflammation by transfer of inflammatory molecules such as cytokines [63, 64], as well as specific moieties that can regulate the inflammatory pathways in target cells [65–67]. COVID-19 is characterized by altered profile of pro or anti-infammatory molecules in EVs derived from patient plasma compared to healthy controls [59–61]. Interestingly, these EVs carrying proinflammtory mediators when delivered on target cells in vitro act as propagators of inflammation [12, 59, 61, 62]. However, knowledge regarding the impact of inflammatory EVs on systemic inflammation using animal models is limited. Additionally, studies analysing the mechanism of loading of the inflammatory protein in EVs combined with their specific cell of origin might shed light on the immunomodulatory role of EVs in COVID-19 and their translation into therapeutic vehicles/targets.

### Extracellular vesicles in COVID-19 associated coagulopathy

An interplay between inflammation and coagulation leading to thrombo-inflammation is critical in the pathogenesis of COVID-19 and helps determine the severity and mortality of the disease [68]. Thrombo-inflammation is characterized by activation of platelets and formation of activated complexes with neutrophils and monocytes. These complexes trigger activation of endothelium, and dysregulation of coagulation, complement and inflammatory response, and activate recruitment of leukocytes [69, 70]. COVID-19 patients displayed higher EV levels derived from different sources such as platelets, endothelial cells, leukocytes, neutrophils, and alveolar macrophages, than controls [8, 14, 71–74]. SARS-CoV-2 can be taken up by platelets in an ACE2 dependent or independent manner [75]. Using transmission electron microscopy macroparticle mediated internalization of SARS-CoV-2 in platelets in an ACE-2 independent manner has been reported [75]. Further, SARS-CoV-2 infection leads to platelet apoptosis and necroptosis and associated morphological changes causing the release of heterogenous population of EVs such as microparticles of different sizes, migrasomes originating form migrating cells, small EVs and large vacuoles [75]. Phosphatidyl serine is a marker of dying cells and activated platelets and an indication of platelet function during COVID-19 [76]. Phosphatidyl serine positive platelet EVs were elevated in COVID-19 patients compared to healthy controls, and strongly correlated with disease severity [74]. The phosphatidyl serine positive platelet-EVs carrying PD-L1 (Programmed death- ligand 1) were shown to preferentially bind to CD8^+^ T cells, [74]. Hence, phosphatidyl serine expressing platelet derived EVs might reprogram the T cell response in COVID19 contributing to disease severity [74]. In contrast, another study reported that total EVs from platelets increase in severe and nonsevere disease compared to healthy controls, but phosphatidylserine-exposing platelet EVs were significantly increased only in non-severe patients [8].

Activated platelets stimulate the surface expression of tissue factor (TF) in monocytes and activated monoctyes release TF in free form and associated with EVs [77]. Furthermore, the assembly of TF with factor VIIa leads to formation of prothrombinase complex in activated platelets promoting thrombin formation and further platelet activation [70]. Several studies reported that circulating EVs TF activity was higher in COVID-19 patients compared to healthy controls and was associated with the severity and mortality of disease [78–80]. Presence of high levels of EVs with TF in severe COVID indicates the ability of EVs to transfer TF intercellularly or directly contribute to thrombus formation and serve as a link between inflammation and coagulation [80]. The coagulopathy induced by the hyperactivate platelets and thrombin formation causes activation of endothelial cells [81]. Endothelial damage/dysfunction is a classical pathological feature in COVID-19, which could be mediated by the immunocoagulopathy or cytokine storm [82, 83]. E selectin expression in endothelial cells mediates the leucocyte infiltration into inflammatory loci leading to tissue or organ damage [84]. In corroboration with this, endothelial EVs in COVID-19 express high levels of E-selectin (CD62), which correlated with critical disease and mortality [85]. In addition, this study evaluated the predictive value of CD62E + EV subtype on COVID-19 related mortality [85]. In addition to being markers of endothelial damage, EVs can act as mediators of endothelial dysfunction [86]. For example, a study by Lascano et al. evaluated the circulating levels of EV-associated neutrophil elastase activity and identified the correlation to pulmonary endothelial damage [87]. The authors suggest that neutrophil derived EVs with high neutrophil elastase activity exacerbate endothelial dysfunction in COVID-19 [87]. As previously mentioned proteins of inflammatory and coagulation pathways are upregulated in EVs in COVID-19 and EV mediated transfer of proinflammtory molecules could induce apoptosis of endothelial cells [59]. Hence EVs are critical mediators of thromboinflammation and mediate vascular damage in COVID-19.

**Fig. 1:**
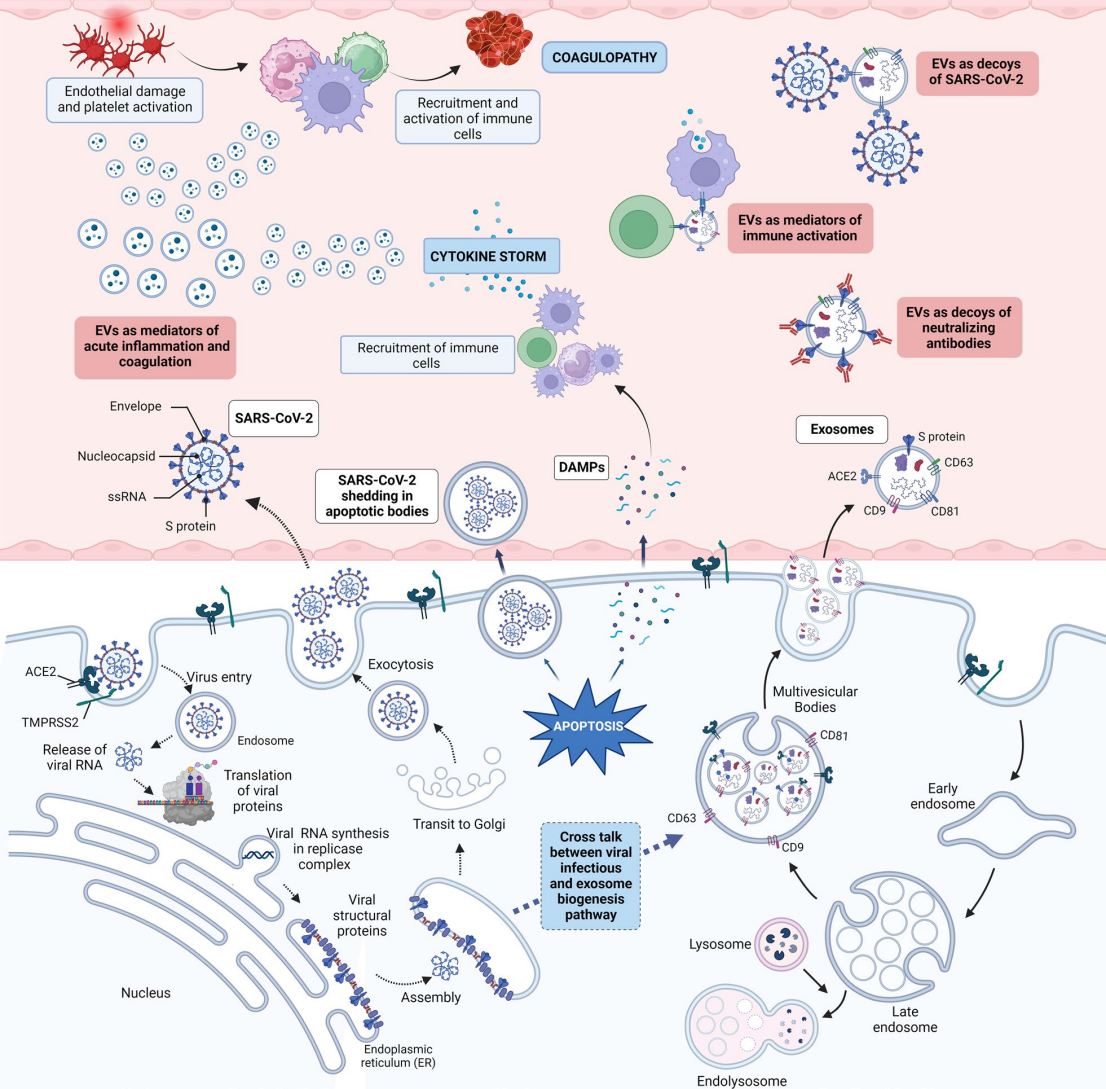
Role of EVs in the pathogenesis of acute COVID-19. The similarities in the EV biogenesis and SARS-CoV-2 infection pathway leads to packaging and release of viral proteins in EVs. The S protein loaded in EVs act as decoys for neutralizing antibodies and contradictorily activate immune response. ACE2 in EVs act as decoys for the virus. The virus induced cellular apoptosis triggers release of virus encapsulated in apoptotic bodies which help the virus to evade immune cells and neutralizing antibodies as well as provide receptor independent viral uptake in target cells. The Damage Associated Molecular Patterns (DAMPs) released by apoptotic cells activate immune cells and release pro-inflammatory cytokines which in adverse conditions lead to cytokine storm. The endothelial damage and platelet activation in SARS-CoV-2 infection leads to blood clot formation and coagulopathy. EVs released from immune cells and platelets are key mediators of imfalmmation and coagulation by transfer of molecular cargo between cells

The majority of studies linking EVs to COVID-19 pathogenesis focus on molecules associated with inflammation and coagulation in EVs and fewer studies have explored into EV moieties other than protein. One such study is by Wang et al. which demonstrated that the expression of miRNAs, miR-7-5p, miR-24-3p, miR-145-5p, and miR-223-3p in circulating EVs to be higher in younger patients and lower in older adults and those living with diabetes. These EV associated miRNAs can effectively inhibit the viral S protein and replication, and contributes to difference in disease severity between patients with different age and comorbidities A study conducted by Song et al., 2020 performed comprehensive analysis of the plasma metabolome and lipidome in COVID-19 patients [88]. This study identified that the lipodomic profile in EVs in COVID-19 reflects the overall plasma lipidomic signature. EVs in COVID-19 were enriched in sphingomyelins and monosialodihexosyl gangliosides(GM3) and had reduced levels of diacylglyerols. The levels of GM3-enriched EVs elavated with disease severity. GM3 levels in plasma of the COVID patients negatively correlated with CD4 + T cell counts implicating the role of GM3-enriched EVs in the regulation of T cell and contribution to disease severity [88]. Clinical translation of the knowledge about the molecular cargo in EVs and their role in the pathogenesis of COVID-19 could help stratification of patients and personalization of therapies. An illustration depicting role of EVs in the pathogenesis of COVID-19 is presented as Fig. 1.

## Extracellular vesicles in diagnosis, therapeutics and vaccine development in COVID-19

The molecular content of EVs reflect the pathological events in their cells of origin, EVs have been evaluated for their biomarker potential to detect the COVID-19 as well as predict the severity of acute disease. Balbi et al., reported that serum-derived EVs from COVID-19 ( +) patients express higher surface markers such as CD49e, CD206, CD86, CD133/1, CD69, CD142, and CD20 than healthy controls (COVID-19 (−) patients) [89]. Using a multivariate model and ROC analysis, it was shown that the expression of tissue factor CD142-bound EV activity is increased in COVID-patients and associated with TNF-α serum levels [89]. It has been showed that severe patients' sera contained increased amounts of CD13 + and CD82 + EVs which correlated with the frequency of IL-6 producing monocytic myeloid-derived suppressor cells. In addition, sera of mild COVID-19 patients contained more HLA-ABC + EVs than healthy controls and more CD24 + EVs than that observed in severe COVID-19 patients [90]. Fujita et al., reported that EV-protein COPB2, a subunit of Golgi coatomer complex, is highly abundant in mild patients compared to severe or critical patients, and evaluated the diagnostic performance to patient stratification in the early phase of disease [91].

In addition, analysis of EV cargo showed that potential biomarkers of COVID-19 related inflammation and coagulation such as fibrinogen, fibronectin, complement C1r subcomponent, and serum amyloid P-component were differentially expressed and effectively discriminated SARS-CoV-2 infection from healthy controls [11]. The standard routine diagnosis of COVID-19 is performed by quantitative PCR (qPCR) of SARS-CoV-2 RNA in respiratory samples [92]. The presence of SARS-CoV-2 in circulation can be detected by qPCR of circulating SARS-CoV-2 RNA but with lower sensitivity [93]. Interestingly, SARS-CoV-2 RNA was identified in small EVs [11]. Detection of SARS-CoV-2 RNA encapsulated in EVs is an attractive alternative to plasma free circulating SARS-CoV-2 RNA which is diluted and degraded by nucleases and exhibits poor performance with quantitative PCR. Ning et al., captured plasma derived EVs using the CD81 antibody and amplified the SARS-CoV-2 RNA by fusing with liposomes loaded with target amplification reagents, enabling accurate identification of COVID-19 patients with greater sensitivity [13]. This can be utilized in situations in which detection of SARS-CoV-2 in blood is required as a secondary test to standard testing using respiratory samples.

In view of the important roles of EVs in COVID-19 pathogenesis, several groups have explored the possibility that these recent findings may aid in the development of new therapeutic interventions against COVID-19. Engineered EVs exposing the ACE2 receptor can act as effective decoys for the SARS-CoV-2 virus and can be utilized for viral neutralization [15, 18, 54, 57, 94–96]. For example, Xie et al., reported that EVs enriched with palmitoylated ACE2 reduced viral load and lung inflammation in human ACE2 transgenic mice [15]. Similarly, ACE2-overexpressing microparticles administered intranasally in a SARS-CoV-2-infected mouse model inhibit the proinflammatory phenotype of alveolar macrophages by increasing lysosomal pH, thus inducing a therapeutic effect against SARS-CoV-2 infection [97]. EV-decorated nanoparticles expressing the ACE-2 receptor was used to compete with ACE2 expressing cells for binding to viral S protein [95]. EVs derived from ACE-2 overexpressing lung cells when administered intranasally were taken up by alveolar macrophages and enhanced lysosomal degradation of the virus by altering the pH levels in the endosomal compartments, leading to increased treatment efficacy in a mouse model [98].

On the other hand, expressing the viral S protein in EVs for specifically targeting cells that highly express ACE2, and delivering anti-viral therapeutic agents has been a promising strategy. For example, engineered EVs loaded with the receptor- binding domain (RBD) of the S protein accumulate specifically in the tissues enriched with the ACE2 receptor, including lungs, and delivered anti-viral siRNAs to suppress infection in vivo [99]. Engineered EVs decorated with the RBD of viral S protein, and labelled with an imageable molecule is an attractive method to monitor the in vivo biodistribution, and the binding affinities of different viral variants to inform therapeutic strategy [100].

Additionally, engineered EVs have been used for targeted treatment of the acute inflammation and organ damage associated with the pathological sequelae of COVID-19. Activated specialized tissue effector EVs derived from genetically modified fibroblasts is reported to inhibit the mTOR pathway and exert cytoprotective and antiviral effects [101]. Also, the therapeutic effect of mesenchymal stem cell derived EVs in regulating the immune response has been evaluated [21]. Interestingly, ginger exosome-like nanoparticle containing miRNA aly-miR396a-5p inhibits the viral Nsp12 and S protein genes and alleviates the lung inflammation in SARS-CoV-2 infection [17].

**Fig. 2:**
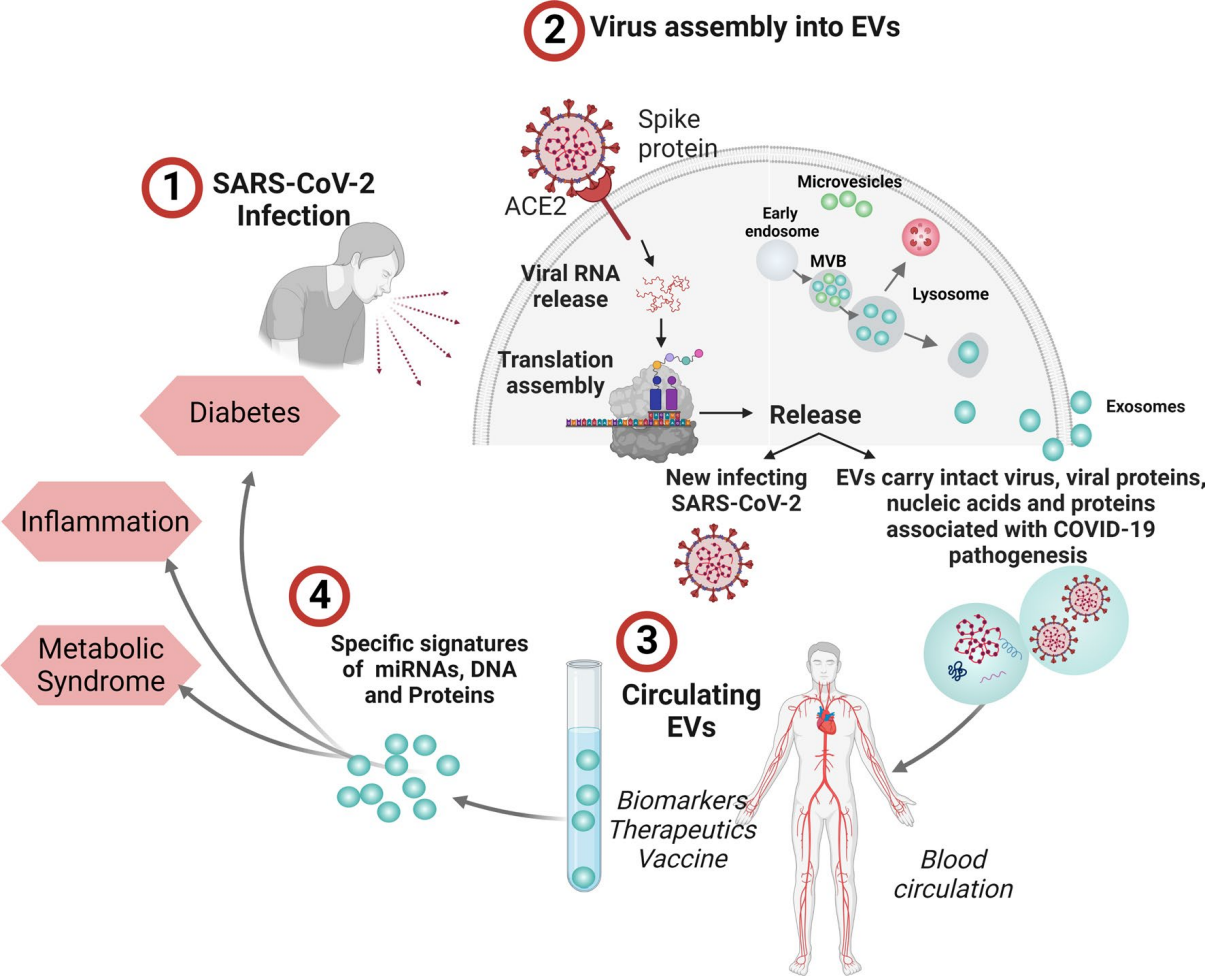
EVs in the pathogenesis of COVID-19 and their utility in clinical translation. EVs released from infected cells may contain SARS CoV 2 virus in double membrane vesicles, viral nucleic acids, proteins, virus induced cellular factors, mediators of inflammation, coagulation and organ damage,and factors associated with perpetuation of COVID-19 or long COVID. The intercellular transfer of cargo via EVs promote the spread of the virus and reprogram host disease susceptibility leading to the development of acute and long term consequences of COVID. EV associated molecular signature in COVID-19 can be investigated for their biomarker potential and therapeutic utility

mRNA-based vaccines directed against the SARS-CoV-2 S protein has been authorized for vaccination in COVID-19 [102, 103]. EVs can be involved in the development of the immune response following immunization. It has also been shown that in healthy adults who received the Pfizer–BioNTech vaccine, EVs carrying the SARS-CoV-2 S protein were detected before the antibody response to S protein [55]. Mice immunized with these EVs developed a specific humoral and cellular immune response to the SARS-CoV-2 S protein [55]. Interestingly, EVs can act as carriers of the vaccine candidate to be delivered in the host cell. For example, EVs loaded with mRNAs encoding immunogenic forms of the SARS-CoV-2 S and N proteins can effectively induce a dose-dependent anti-S and anti-N antibody response as well as antigen-specific T cell-mediated immunity in mice [24]. In another study Wang et al., showed that an inhalable COVID-19 vaccine composed of human lung-derived EVs decorated with a recombinant SARS-CoV-2-RBD protein induces, in mice and hamsters, both a humoral and cellular immune response against SARS-CoV-2 infection and can be stored at room temperature [23]. On the other hand, DNA vectors expressing the fusion products of inactive HIV-1 Nef protein and SARS-CoV-2 S and N proteins loaded in EVs induces a strong CD8 + T cell immunity in mice immunized with these vectors [26, 104, 105]. An illustration depicting the clinical utility of EVs in COVID-19 is presented as Fig. 2.

## COVID-19 and long-term effects

Long COVID or post acute sequelae of COVID-19 is a condition affecting multiple organs comprising of multitute of symptoms that follows the acute infection from SARS-CoV-2 [106]. WHO describes long term effects of COVID-19 otherwise named ‘long COVID’ as the continuation or development of new symptoms 3 months after the initial SARS-CoV-2 infection with these symptoms lasting for at least 2 months with no other explanation [107]. Fatigue, shortness of breath and cognitive dysfunction are amongst the most frequent symptoms of long COVID, although more than 200 different symptoms can be associated with the condition [107]. A pioneering study conducted in COVID-19 patients from Italy reported the persistence of at least one COVID-19 related symptom 60 days after recovery. The most common symptoms were fatigue and dyspnoea, and joint pain and chest pain were found in high proportions [108]. This delay in returning to the former heath trajectory is observed not only in patients that have recovered from severe COVID-19 which required intensive care admissions, but also in patients recovered from mild and moderate symptoms [109] Furthermore, many studies reported patients showing variety of symptoms in the post-acute phase affecting multiple organs including lungs, heart, immune system, nervous system, gastrointetsinal system, kidneys and endocrine system [106]. Around 10% of people who have had COVID-19 have reported long COVID, however the exact numbers are uncertain [106]. In addition, studies to understand the pathophysiology of long COVID-19 are at an early stage. Table 1 summerizes the major studies that reported long COVID as persistence of symptoms or onset of new sympstoms in patients post acute illness.

**Table 1:** Summary of studies on long term effects of COVID-19

No	Study design	Findings	References
1	Analysis of peripheral CD4 T cells from 13 patients confirmed negative of SARS-CoV-2 for 2 to 4 weeks	CD4 + T cell spectrum and inflammatory cytokine profile are altered in convalescent patients. Virus-specific IgG- or IgA-identified in severe patients	[188]
2	Prospective study in patients (n = 100) after 71 days of recovery from COVID	Cardiac magnetic resonance imaging identifies cardiac involvement and myocardial inflammation in long COVD independent of disease severity and presence of co-morbidities	[125]
3	Prospective observational study in patients (n = 143) for 60 days after onset of the first COVID-19 symptom	Significant proportion of individuals reported persistence of symptoms such as fatigue, dyspnea, joint pain chest pain	[108]
5	Prospective observational study in patients (n = 100) for 104 days after symptom onset	Reported impairment of pulmonary diffusion in long COVID and association with the most severe SARS-CoV-2 cases	[115]
6	Retrospective study using electronic health database upto 6 months after COVID-19 diagnosis	Broad array of pulmonary and extrapulmonary clinical manifestations were identified in post acute COVID patients	[127]
7	Prospective observational study in patients (n = 113) for 4 months after COVID-19	Identified functional and radiological abnormalities in lungs due to small-airway and lung parenchymal disease	[119]
8	Prospective longitudinal study in patients (n = 33) for 6 months post COVID	Reported impaired pulmonary function and structure in long COVID	[116]
9	Prospective longitudinal study in patients (n = 1276) up to 1 year post COVID	Most survivors had a good physical and functional recovery during 1-year follow-up, and had returned to their original work and life. Pulmonary abnormalities persisted in some critically ill patients	[189]
10	Prospective longitudinal study in patients (n = 834) at 4 months post COVID	Reported persistence of symptoms and lung-scan abnormalities post-COVID	[190]
11	Prospective study of 442 and 353 patients over four and seven months respectively after symptom onset	Symptoms including shortness of breath, anosmia, ageusia or fatigue even in non-hospitalised patients was observed at four and seven months post-infection	[112]
12	Prospective longitudinal study in patients (n = 199) up to 6 months after discharge	The prevalence of persistent symptoms following hospitalization in long COVID reported	[122]
13	Prospective longitudinal study in patients (n = 135) at 3 months, 6 months, 9 months, and 12 months after hospital discharge	In a sub-group of patients who recovered from severe disease evidence of persistent physiological and radiographic change reported	[111]
14	Observational study using questionnaires in COVID positive (n = 4231) and controls (n = 8462) for 90–150 days after COVID-19 diagnosis	Persistence of symptoms such as chest pain, difficulties with breathing, pain when breathing, painful muscles, ageusia or anosmia, tingling extremities, lump in throat, feeling hot and cold alternately, heavy arms or legs, and general tiredness defined post-COVID condition	[191]
15	Retrospective study based on electronic health database followed for 30–365 days after the diagnosis of COVID-19	The occurrence of 26 clinical conditions involving multiple organ systems were significantly higher in COVID recovered patients than controls	[192]
16	Retrospective study based on electronic health database followed for 6 months after the diagnosis of COVID-19	People who had COVID showed increased mortality and post-acute sequelae in pulmonary and extrapulmonary systems than matched controls. The death and post-acute sequelae in infected people who had prior vaccination was lower than infected people without vaccination	[170]
17	Retrospective study based on electronic health database followed for 12 months after the diagnosis of COVID-19	Individual with COVID-19 are at increased risk of cardiovascular disease and associated with the severity of the acute disease	[178]
18	Retrospective study based on electronic health database followed for 352 days after positive COVID test	COVID-19 patients exhibited an increased risk and excess burden of incident diabetes and antihyperglycemic use. Risk of diabetes increased in a graded manner depending on the severity of the disease	[135]
19	Prospective study in COVID patients who developed new and persistent shortness of breath for > 3 months post-recovery (n = 41) were evaluated for 8.9 ± 3.3 months after COVID	Performed cardiopulmonary exercise testing in patients. Circulatory impairment in exercise is reported as a post-acute sequalae and cause of unexplained dyspnea	[193]
20	Prospective observational study in COVID positive and negative individuals with and without encephalomyelitis/chronic fatigue syndrome (n = 61) for 6 months after diagnosis	COVID-19 leads to persistent fatigue syndromes in a subset of individuals following mild to moderate infectious disease	[194]
21	Retrospective pilot study of COVID positive patients (n = 63) for 12 months post infection	Measured the concentration of SARS-CoV-2 S and N proteins and panel of cytokines. Post acute sequelae is associated with persistence of circulating S protein	[195]
22	Retrospective pilot study in COVID positive patients (n = 147) with and without long COVID 8 months after infection	Inflammatory mediators (IFN-β, PTX3, IFN-γ, IFN-λ2/3 and IL-6) are associated with long COVID and evaluated their biomarker potential	[146]
23	Prospective study in COVID positive patients (n = 106) and non-COVID controls (n = 85) up to 6 months after infection	Reported alteration in gut microbiome in patients with long term COVID	[196]
24	Retrospective study from South African Long COVID/PASC registry (n = 845) Blood sample collection from 80 patients	Long COVID is associated with platelet hyperactivation and microclot formation and platelet and clotting grading system can be used for the early detection of long COVID	[149]
25	Prospective observational study (n = 58) with COVID patients with and without long COVID and healthy controls for 18 months after infection	Long term effect of COVID-19 is associated with persistent capillary rarefication	[197]
26	Prospective, longitudinal in COVID positive patients with long COVID (n = 534) for 1-year post-infection	Cardiac impairment is present in long COVID and persist upto 12 months	[198]
27	Retrospective study based on electronic health database from 30 days to 2 years after COVID-19	Long COVID is associated with increased risk of kidney outcomes	[199]
28	Retrospective study based on electronic health database for 2 years after COVID-19	Long COVID is associated with persistence of psychotic disorder, cognitive deficit, dementia, and epilepsy or seizures	[200]

### Pulmonary consequences of COVID-19

Impaired lung function characterized by decreased diffusion capacity and diminished respiratory muscle strength is reported to persist 30 days after hospital discharge in COVID-19 patients [110]. A study by Wu et al., prospectively followed up severe COVID-19 patients for 3, 6, 9, and 12 months after discharge and reported persistence of pulmonary changes at 12 months after discharge. There was a significant decline in diffusion capacity and persistence of radiological changes in a subgroup of patients (24%) at 12 months after discharge [111]. The persistence of long-lasting symptoms, including shortness of breath, is reported even in non-hospitalized patients, up to seven months post-infection [112]. Among the pulmonary function tests, impairment in diffusion capacity is reported most commonly followed by ventilatory defects in COVID-19 survivors, mostly in association with the severity of the disease [113–117]. The predominant chest CT abnormality associated with acute COVID-19 is the ground glass opacity of lungs, which peaks during illness and had persisted at the time of hospital discharge [118]. In patients with more severe disease at acute phase, the persistence of chest CT abnormalities with ground glass opacity were reported in studies that were performed up to 6 months follow up [111, 116, 119–122].

### Cardio-metabolic consequences of COVID-19

The long-term impact of COVID-19 involves abnormalities associated with the cardiovascular system as well [123]. Myocardial inflammation (myocarditis) has been described in severe acute COVID-19 with activation of DNA damage [123, 124]. A study reporting cardiac magnetic resonance imaging in recovered COVID-19 patients around 71 days after COVID-19 diagnosis identified cardiac involvement and ongoing myocardial inflammation independent of COVID-19 severity, or course of acute illness and presence of co-morbidities [125]. Moreover, patients with mild or no symptoms that were diagnosed as SARS-CoV-2 positive by real-time PCR showed myocarditis nearly 12–53 days after diagnosis [126]. Retrospective analysis of US electronic health database identifies that COVID-19 diagnosis is associated with increased incidence of hypertension, chest pain, coronary atherosclerosis and heart failure compared to healthy controls and changes in a graded manner according to the severity of the disease [127]. However, the pathophysiology of long term impact on COVID-19 on cardiovascular system is currently poorly understood.

Co-morbidities such as hypertension, cardiovascular diseases, obesity, and insulin resistance, are responsible for poorer prognosis of acute COVID-19 [123, 128–130]. Hyperglycaemia is associated independently with COVID-19 related death in people with type 1 and type 2 diabetes [128]. Furthermore, in COVID-19 patients with type 2 diabetes, well-controlled blood glucose is correlated with reduced mortality and adverse outcomes [129].

The pathophysiology of COVID-19 and diabetes has a bidirectional interplay in which COVID-19 could induce metabolic complications including diabetic ketoacidosis and hyperglycaemia in diabetic patients [130]. In addition, COVID-19 can precipitate newly diagnosed diabetes, leading to long-term metabolic consequences in recovered patients [131]. This may be the result of unresolved inflammation from the acute SARS-CoV-2 infection which correlates with the persistence of insulin resistance and abnormal beta cell function [131]. Pancreatic beta cells express the ACE2 receptor protein, the TMPRSS2 enzyme protein, and neuropilin 1 (NRP1), all of which may help the entry of SARS-CoV-2 into beta cells [132]. Infection of pancreatic beta cells with SARS-CoV-2 can lead to cellular reprogramming, cell death, and reduced production and release of insulin from pancreatic beta cells [133]. Rathmannn et al. reported that individulas with COVID-19 showed increased incidence of type 2 diabetes compared to controls with acute upper respiratory tract infections up to 500 days post infection [134]. Retrospective study using the electronic health database of the US Department of Veterans Affairs in a cohort of 181280 COVID positive participants and contemporary (n = 4118441) and historical (n = 4286911) controls for 352 days post-COVID reported increased burden of diabetes and anti-hyperglycemic use [135]. However, retrospective study using national register in Scotland in a cohort of 365,080 individuals reported type 1 diabetes increased during pandemic, but was not associated with SARS-CoV-2 infection [6]

### Other post-acute sequelae of COVID-19

Long COVID can impact multiple organs and a multitude of symptoms have been identified [106]. Chronic health loss in COVID-19 survivors involving multiple organs, including nervous, gastrointestinal, haematological, and musculoskeletal systems have been reported [127]. During acute illness, SARS-CoV-2 may enter brain tissue and invade the olfactory nerve causing anosmia [136]. Headache, vertigo and other symptoms associated with chemosensory dysfunction and cognitive symptoms are reported to persist after COVID-19 [108, 112, 137]. In addition, psychiatric problems associated with COVID-19 are reported to persist months after recovery and this includes post-traumatic stress disorder, depression, anxiety, and insomnia [138]. Renal sequelae of COVID-19 is characterized by high mortality, which was reported in a follow up study of COVID-19 survivors with acute kidney disease requiring renal replacement [139].Gastrointestinal sequelae include gut dysbiosis, abdominal pain and nausea whilst in the reproductive system, long COVID leads to erectile dysfunction and reduced sperm count in men, and abnormal premenstrual symptoms and irregular menstruation in females [106]

In children, COVID-19 was initially reported to cause only mild disease without severe manifestations [140]. However, in April 2020, a severe systemic hyperinflammatory condition called multisystem inflammatory syndrome (resembling Kawasaki disease), that develops 4–6 weeks after COVID-19, was reported in children. This involves multiple systems, and clinical symptoms such as fever, a hyperinflammatory state, skin lesions, abdominal pain, diarrhoea and vomiting [141]. Cardiac manifestations are predominant, including myocarditis, coronary artery abnormalities, pericarditis, pericardial effusion, and valvular regurgitation [142]. Taken together, the long-term sequelae of COVID-19 in recovered patients can seriously threaten the overall wellbeing and day to day activities. Hence, studies exploring biomarkers to predict the occurrence of long COVID is critical for patient management.

There are several hypotheses regarding the underlying mechanism of long COVID, including presence of a long-lasting inflammatory response from the acute disease that affects multiple organs, tissue damage during acute disease resulting in release of autoantibodies, microbiome dysbiosis in gut which may cause chronic inflammation, reactivation of latent viruses such as Epstein Barr virus, persistence of viral replication and all Pathogen Associated Molecular Patterns (PAMPS) after the resolution of the acute infection, and continuation of tissue damage that occurred during active viral replication and acute disease [143–148]. An example for continuation of tissue damage is that significant micro clot formation that are resistant to fibrinolysin in the acute disease was continued to the post COVID phase [149, 150]. It is possible that some, all or none of these mechanisms drive the pathogenesis of long COVID. Indeed given the diversity of clinical symptoms in long COVID patients it is possible that different mechanisms of disease drive different clinical manifestations of the illness.

## EVs as mediators of long-term complications of COVID-19

The long-term consequences of COVID-19 or long COVID-19 is an unexplored area that urgently needs studies to understand the clinical trajectory and biological mechanisms associated with the complications. Patients with long COVID have activated innate immune cells, reduced naïve T and B cells and elevated levels of proinflammatory cytokine including interferons persisting more than 8 months following convalescence [146]. This study may indicate that the virus or viral antigen might be persisting post-acute phase in COVID [146]. The ability of EVs to maintain the viral infection and help viral establishment in chronic latent infections has been reported [151]. For example, in Human Immunodeficiency Virus (HIV) infection, EVs from infected cells transfer viral components to uninfected cells and prime the neighbouring cells to viral infection [152, 153]. EVs help maintain a favourable host environment for long periods of time when the viral replication is minimal and promote persistent viral infection [151]. However, direct evidence regarding persistence of SARS-CoV-2 infection in long COVID patients is lacking. Heterogenous populations of EVs play a variety of roles as mediators of inflammation in diseases [154–156]. Studies investigating the possible roles of EVs in sustenance of inflammation and programming of host immune response in SARS-CoV-2 infection will provide more insights into this.

Nevertheless, EVs could have possible roles in sustaining the pathological effects/tissue damage caused by SARS-CoV-2 and promoting the persistence of disease. For example, EVs from COVID-19 patients 3-months post acute phase had differential protein profile associated with inflammation, coagulation and liver function [157].EVs from different stages of COVID-19 infection, including pre-symptomatic, disease, and convalescent phase, reported alterations in protein and lipid composition during the course of disease [158]. Specifically, alterations in EV protein and lipids associated with immune response, coagulation processes and cholesterol metabolism were identified along the temporal trajectory of the disease [158]. Further, EVs from different stage of infection produced distinct metabolic effect on target cells indicating EVs as mediators of metabolic dysregulation in COVID-19 [158]. However, no studies so far have explored into the role of EVs in perpetuating the metabolic dysregulation of COVID in long COVID. Interestingly, EVs and their molecular cargo have been described as prognostic biomarkers and mediators of intracellular signalling in metabolic diseases [159–161]. Diabetes and obesity are associated with alterations in the concentration, cellular origin, and molecular cargo of circulating EVs, which affect target organs at a systemic level, suggesting a potential pathogenic role of EVs in metabolic diseases [159–161]. Similarly, circulating EVs are increased in cardiovascular diseases [162, 163], with altered molecular cargo [164–166], and involved in cellular crosstalk between cardiomyocytes and non-cardiomyocytes [167], contributing to the pathology of cardiovascular diseases [168, 169]. Given the strong association of EVs to endocrine-related conditions [7] and their ability to act as metabolic effectors in COVID-19 [158] they could have possible roles in mediating the cardio-metabolic sequelae of long COVID.

Postacute neurological sequelae of SARS-CoV-2 infection includes an array of neurological disorders ranging from cognitive or memory impairment to stroke, encephalitis or encephalopathy [170]. However, the pathological basis of these neurological manifestations which are continuing or newly onset after many months of acute infection is not clear. Interestingly, long COVID with neurological symptoms is associated with altered profile of CD4 + and CD8 + T cells and B cells in cerebrospinal fluid which might indicate the role of sustained inflammation in this condition [171] In addition, long COVID patients with neurological symptoms showed differential expression of plasma cytokines compared to recovered patients without neurological symptoms [172]. EVs of neuronal origin can be specifically isolated using antibodies against L1 cell adhesion molecule (L1CAM) which is expressed in neurons [172, 173]. Interestingly, SARS-CoV-2 S and N protein were higher in neuronal EVs in COVID-19 in post acute phase (both with and without long COVID) compared to healthy controls [173]. Neuron derived EVs from recovered individuals with post-acute neuronal synptoms showed differential expression of mitochondrial proteins associated with metabolism, energy generation, ion channels and neuronal survival [173]. However, the link between mitochondrial dysfunction caused by SARS-CoV-2 with implications on central nervous system is not clearly known. A study by Sun et al. reported increased expression of protein markers of neurodegeneration including amyloid beta, neurofilament light, neurogranin and tau in neuron EVs in patients with long COVID at 1 to 3 months post-infection [172]. This is in line with the pivotal role of EVs in the pathogenesis of neurodegenerative diseases transfer of pathological proteins between neurons [174–176]. Overall, current evidence linking EVs to the post-acute sequelae of COVID-19 is limited a. Large cohort studies analysing the cargo of total or cell type specific EVs across the disease trajectory upto to the long-COVID stage combined with mechanistic studies to understand the effect of EVs on recipient cells are required to shed light into this area of research. Figure 2 illustrates the potential involvement of EVs in the pathogenesis of COVID and their utility in clinical translation.

## Future directions of EV research for long COVID

Currently there are several unanswered clinical questions in the long-term consequences of COVID-19 which are (1) How many patients recovering from COVID will suffer from long-term consequences? (2) What are the underlying disease conditions that contribute to development of long term sequelae? (3) Can we predict the development of long COVID during the acute phase of the disease? (4) How can long COVID be supported, medically or using life style interventions? [109, 146, 177]. Identification of biomarkers that can predict long COVID in acute phase is a critical research need. It is known that certain factors such as severity of the acute infection, vaccination status, sex and co-morbidities predict the risk for long COVID [106, 143, 178] but there is a lack of biomarker that can predict ones risk of long COVID. This impairs patient diagnosis and seriously affects the estimates of disease prevalence and subsequent public health planning.

However, obtaining study participants in a longitudinal study design for extended time periods and resources required for conducting the patient recruitment, sample collection and processing pose major challenge in conducting large scale multi-centre studies. Long COVID is characterised by a diverse array of symptoms possibly originating from different mechanisms of disease and currently there is an absence of clear diagnostic criteria causing difficulty in interpreting findings between different studies. A clear definition of patient inclusion criteria with definition of outcomes and common protocols is required to overcome this [109]. In addition, the potential bias attributed to the variant of SARS-CoV-2 infection, vaccination status and treatment undergone duing acute disease should be considered [179, 180].

When using EVs in clinical studies, strigent and uniform methods for collection and storage of samples, isolation of EVs, analysis of contents and reporting of results should be employed. EV levels and contents are highly dynamic and change drastically with pre-analytical variables such as time of processing, temperature of storage and processing, presence/absence of coagulation, degree of hemolysis etc. [181]. For example, EVs derived from non-target cells such as blood cells at the time of sample collection could mask the disease specific EV signature from target cells [182]. In addition, different EV isolation methods yield EVs with different purity and particles/factors coisolated with EVs can mask the disease signature or contribute to the function of EVs depending on the research question being addressed [181]. Hence straight forward and robust method of EV isolation and EV characterization should be employed for optimum results in clinical translation. Proteins and miRNAs are the most studied molecules in EVs due to advancement in proteomics and sequencing techniques [183, 184]. However, mRNA [185], DNA [186] and lipid content [187] of EVs could be critical mediators of disease mechanisms and required to be studied with regard to long COVID. Further studies to address the limitations of EV research in large scale clinical studies could help overcome the potential bias attributed by the above factors and develop a comprehensive understanding of EVs in long COVID.

## Concluding remarks

As we navigate the multifaceted landscape of COVID-19's impact on human health, it becomes evident that our journey towards understanding and addressing its long-term consequences is far from over. Longitudinal observational studies and rigorous clinical trials stand as indispensable pillars in constructing a comprehensive comprehension of the virus's enduring effects. These efforts not only empower us to decipher the intricate aspects of COVID-19’s influence on patients' well-being but also provide the insights needed to tailor healthcare systems to effectively manage these consequences. In this context, the intriguing role of EVs and their intricate molecular cargo emerges as an area of great promise. By functioning as potent mediators of intercellular communication in various metabolic disorders, EVs could potentially establish a crucial link between COVID-19 and its enduring aftermath. The unique molecular signature (e.g., bioactive lipids, proteis and nuclic acids) carried by EVs during the acute phase of the disease unveils the prospect of employing them as sensitive indicators of the long-term implications of COVID-19, potentially offering a valuable tool for prognosis and monitoring. However, the significance of these insights extends beyond diagnostic applications. Particularly the potential for EVs to serve as precursors of novel therapeutic targets for patients with long COVID. In providing fresh perspectives into the molecular pathways of EV signaling within the pathogenesis of these enduring consequences, we uncover the possibility of discovering previously uncharted roads for intervention. This presents optimism for the significant population dealing with the complexities of long COVID, offering the potential for improved management and enhanced quality of life.

It is important to acknowledge the limitations of our current understanding. The field of EV research in the context of COVID-19’s long-term effects is still evolving, and while the potential is promising, further comprehensive longitudinal studies are required to validate the role of EVs as both diagnostic indicators and therapeutic avenues. Moreover, the intricate interplay between EVs and the numerous factors influencing long COVID underscores the requirement for a comprehensive exploration that fully embraces the multifaceted nature of this condition. The integration of EV research into our understanding of COVID-19's long-term implications not only enriches our scientific understanding but also holds the promise of translating knowledge into tangible benefits for individuals affected by this widespread and intricate condition. Finally, the collective efforts of researchers, healthcare professionals, and institutions remain crucial in unraveling the problem of long COVID.

## Data Availability

Not applicable.
